# Prognosis evaluation of universal acute coronary syndrome: the interplay between SYNTAX score and ApoB/ApoA1

**DOI:** 10.1186/s12872-020-01562-6

**Published:** 2020-06-15

**Authors:** Xiaotong Wang, Zhongyu Wang, Bing Li, Ping Yang

**Affiliations:** Department of Cardiology, China-Japan Union hospital of Jilin university, 126 Xiantai Street, Changchun, 130031 China

**Keywords:** SYNTAX score, SYNTAX II score, apoB/apoA1, PCI, Prognosis

## Abstract

**Background:**

Acute coronary syndrome (ACS) is a group of clinical syndromes associated with substantial morbidity and mortality rate. SYNTAX and SYNTAX II score used to be a reference for surgical selection of coronary revascularization and prognosis evaluation in patients with 3-vessel or left main artery disease. In addition, apoB/apoA1 is an important predictor of ACS risk. This study aims to assess the prognosis value of different kinds of SYNTAX score together with apoB/apoA1 in universal ACS patients (Regardless of ACS type, lesion location and vessel numbers).

**Method:**

Three hundred ninety-six patients with ACS undergoing percutaneous coronary intervention(PCI)and coronary stenting from 2013 to 2014 were chosen and recorded the major adverse cardiovascular and cerebrovascular events (MACCE) and quality of life during the next 5 years. According to SYNTAX and SYNTAX II score, the patients were divided into low-risk, medium-risk and high-risk groups, and the clinical features, MACCE incidence and EQ-5D score at each time points were compared. And the predictive factors of MACCE incidence were analyzed.

**Results:**

① Compared with SYNTAX low-risk group, MACCE incidence in 1 year significantly increased in medium/high risk group (*p* = 0.011). Compared with SYNTAX II low-risk group, MACCE incidence in 5 years significantly increased in medium and high-risk group (*p* = 0.032). ② Compared with SYNTAX II low-risk group, cardiovascular mortality in 3 and 5 years significantly elevated in high-risk group (*p* = 0.001, *p*<0.001 respectively). ③ Compared with SYNTAX II low and medium-risk group, EQ-5D score in 5 years significantly decreased in high-risk group (*p* = 0.019, *p* = 0.023 respectively). ④ ApoB/ApoA1 was more likely to be classified as high risk in SYNTAX/SYNTAX II medium and high-risk group (*p* = 0.023, *p* = 0.044 respectively). ⑤ Logistic regression analysis showed that apoB/apoA1 was an independent predictor of MACCE events in hospital and 5 years (*p* = 0.038, *p* = 0.016 respectively), SYNTAX score was an independent predictor of MACCE events in 1 year (medium-risk group: *p* = 0.02; high-risk group: *p* = 0.015) SYNTAX II score was an independent predictor of MACCE events in 5 yeasrs (*p* = 0.003).

**Conclusions:**

① SYNTAX score has a high predictive value for short-term prognosis while SYNTAX II score is more predictive of long-term prognosis. ② SYNTAX II score is superior to SYNTAX score in predicting cardiovascular death. ③ The combination of apoB/apoA1 high-risk and SYNTAX II medium and high-risk group is the focus of clinical treatment and long-term follow-up observation.

## Background

Acute coronary syndrome (ACS) is a group of clinical syndromes characterized by rupture or invasion of coronary atherosclerotic plaque, resulting in complete or incomplete coronary occlusion. ACS including unstable angina pectoris(UA),non-ST-elevated myocardial infarction(NSTEMI)and ST-elevated myocardial infarction(STEMI) is associated with substantial morbidity and mortality rate [1, 2], and thus becomes the foucs of treatment. Coronary revascularization is an effective treatment for acute coronary syndrome [3, 4] including percutancous coronary intervention (PCI), coronary artery bypass grafting (CABG) and hybrid coronary revascularization (HCR). SYNTAX score mainly conducts quantitative analysis according to the anatomical characteristics of coronary artery, such as location, length, stenosis degree, bifurcation, etc., which is a comprehensive assessment method for evaluating the severity of coronary artery lesion [5, 6]. On the other hand, the SYNTAX II score accounts for clinical factors in addition to the coronary artery anatomy.. At present, SYNTAX and SYNTAX II score have been used for the surgical selection of coronary revascularization and prognosis evaluation in patients with 3-vessel or left main artery disease [7, 8]. However, no studies have focused on whether these scores have positive predictive values in the occurrence of MACCE events in universal ACS patients (regardless of ACS type, lesion location and vessel numbers) and whether there is any difference between the two. This prospective study aims to explore the correlation between different SYNTAX scores and prognosis of patients with universal ACS through analyzing the clinical data in hospital and following-up MACCE events and quality of life for 5 years. The scoring system is further enriched by combining with other clinical variables (such as apoB/apoA1, an important predictor of ACS risk) in order to achieve better predictive effect.

## Methods

### Object

The study consecutively enrolled 456 patients diagnosed ACS and underwent PCI and stent implantation in China-Japan union hospital of Jilin university from January 1st, 2013 to January 1st, 2014 regardless of ACS type, lesion location and vessel numbers. We excluded participants if they did not consent to the study (*n* = 34) or if they withdrew from follow-up in 1 year (*n* = 5) or after 1 year (*n* = 21). Therefore, a total of 396 patients including 274 males and 122 females with complete data were included in the final analysis.

Exclusion criteria: (1) Stent implantation was refused or too complicated to conduct (2) Combined with severe hepatic insufficiency (AST and/or ALT>three times the upper limit of normal) and/or renal insufficiency (Serum creatinine>221 μmol/L) (3) Combined with severe infection, trauma or in the recovery of acute infection (4) Combined with tumor (5) Combined with severe anmia(Hemoglobin<60 g/L) and other hematological system diseases (6) Combined with congenital heart disease, valvular heart disease, cardiomyopathy, pulmonary heart disease and aortic dissection.

### Method

#### Data collection

General data such as gender, age, body mass index(BMI), past disease history, personal history, etc. and auxiliary examination including ejection fraction, triglyceride(TG), total cholesterol(TC), low density lipoprotein-cholesterol(LDL-c), non-high density lipoprotein-cholesterol(non-HDL-c), ApoB/ApoA1, hemoglobin(Hb), platelet(PLT), hematokrit(HCT) were collected. Non-HDL-c is defined as the result that subtract HDL-c from TC.

Age is divided into four grades: (1) Young: < 45 years old; (2) Middle-age: 45 to 59 years old; (3) Old age: ≥ 60 years old. According to WHO classification, BMI is defined as low weight when BMI is < 18.5, normal when BMI is 18.5–24.9, pre-obesity when BMI is 25.0–29.9, obesity when BMI is ≧30.0. According to *2016 ESC guidelines for the diagnosis and treatment of acute and chronic heart failure* [9], ejection fraction is divided into the following three grades: (1) ≥ 50%; (2) 49–40%; (3) ≤ 40%. According to INTERHEART research [10], patients from different age groups were defined as high-risk group and low-risk group according to the ApoB/ApoA1 risk prediction criteria:age < 45 years old, ApoB/ApoA1 > 1.76 is defined as high-risk group, ApoB/ApoA1 < 1.76 is defined as low-risk group; 45 years old ≤ age ≤ 55 years old, ApoB/ApoA1 > 1.70 is defined as high-risk group, ApoB/ApoA1 < 1.70 is defined as low-risk group; 56 years old ≤ age ≤ 65 years old, ApoB/ApoA1 > 1.59 is defined as high-risk group, ApoB/ApoA1 < 1.59 is defined as low-risk group; 66 years old ≤ age ≤ 70 years old, ApoB/ApoA1 > 1.52 is defined as high-risk group, ApoB/ApoA1 < 1.52 is defined as low-risk group; age > 70 years old, ApoB/ApoA1 > 1.24 is defined as high-risk group, ApoB/ApoA1 < 1.24 is defined as low-risk group.

#### Coronary artery lesion evaluation

Left and right coronary angiography was performed with Judkins method, and the results were determined by experienced cardiologists. Coronary artery lesions are divided into following parts: ① left main artery(LM), ② left anterior descending ramus proximal segment(PLAD), ③ left anterior descending ramus middle and distal segment(MLAD), ④ Obtuse marginal, ramus circumflexus, left posterior descending ramus, left posterolateral ramus(RCX), ⑤ Right coronary artery, right posterior descending ramus, right posterolateral ramus, acute marginal ramus(RCA), ⑥ Ramus medianus(RAM). According to the angiography results, SYNTAX scoring calculator (http://www.syntaxscore.com) was used to score coronary arteries with diameter ≥ 1.5 mm, taking into account the left and right dominant classification of coronary arteries, lesion site, stenosis degree and pathological features. SYNTAX II score is the combination of SYNTAX score and the clinical variables, which include patient’s age, gender, creatinine clearance rate, left ventricular ejection fraction (LVEF), left main disease, peripheral vascular disease (PVD), chronic obstructive pulmonary disease (COPD).

#### Follow-up procedure

All patients were followed up by telephone, and the incidence of MACCE events at different time points were collected according to the patient’s condition changes and rehospitalization. EQ-5D scores at different time points were calculated through questionnaires to explore whether the quality of life of patients had any changes. The follow-up time points were 1 year, 3 years and 5 years after coronary stent implantation (while the EQ-5D questionnaires were 1 year and 5 years).

MACCE events are defined as composite endpoint events of cardiovascular death, recurrent myocardial ischemia/infarction, recurrent revascularization, new or aggravated heart failure, stroke, or peripheral vascular disease. The EQ-5D score includes six aspects: mobility, self-care ability, daily activity ability, pain or discomfort, anxiety or depression, and self-evaluation of quality of life.

### Statistical analysis

All data in this study were analyzed by SPSS 22.0 software. Kolmogorov-smirnov method was used for normal distribution test. Measurement data following normal distribution were represented by (*x ± s*) and comparison between the two groups was conducted by *t* test, whereas measurement data that didn’t coincided with normal distribution were expressed as median and quartile [*M*(*Q*1-*Q*3)] and comparison was conducted by *Mann Whitney* test. Analysis of variance (ANOVA) was used for comparison among three groups. Enumeration data were expressed by [*n*(%)], and comparison was conducted by chi-square or Fisher’s exact test. Univariate logistic regression analysis was conducted on all variables, and whether the variable was included in the multivariate logistic regression analysis was determined based on *p* results and professional knowledge. The OR value and 95% confidence interval (CI) were further calculated. Bilateral *p* < 0.05 was considered statistically significant.

## Results

### Comparison of clinical baseline data between SYNTAX low and medium/high risk group

Patients are divided into 3 groups according to SYNTAX score [11, 12]: low-risk group (SYNTAX score 0–22), medium-risk group (SYNTAX score 23–32), high-risk group (SYNTAX score ≥ 33). In view of the small number of middle-risk group and high-risk group, the two groups were combined into one group for comparison.

As shown in Table 1, compared with the low-risk group, the proportion of patients with in-hospital heart failure was higher in the SYNTAX medium/high risk group (*p* = 0.021), while there was no statistical difference in the remaining general data. In addition, apoB/apoA1 was more likely to be defined as high-risk in SYNTAX medium/high risk group (*p* = 0.023). Although there was no statistical difference in other serum biochemical items, the mean value of apoB/apoA1 was still higher in the middle-high risk group than the low-risk group.

**Table 1:** Comparison of clinical baseline data between SYNTAX low and medium/high risk group [n(%),M(P_25_--P_75_)]

Item		Low-risk group (0–22)	Medium/high risk group (≥23)	*p*
n		331	65	
Gender	Male	224(67.7)	50(76.9)	0.140
	Female	107(32.3)	15(23.1)	
In-hospital ventricular fibrillation		1(0.3)	0	1.000
In-hospital ventricular tachycardia		2(0.6)	1(1.5)	0.417
In-hospital atrial fibrillation		6(1.8)	3(4.6)	0.170
In-hospital heart failure		52(15.7)	18(27.7)	0.021
Hypertension		211(63.7)	40(61.5)	0.736
Diabetes		62(18.7)	17(26.2)	0.171
CHD history		61(18.4)	10(15.4)	0.559
Myocardial infarction history		22(6.6)	6(9.2)	0.432
PCI history		18(5.4)	6(9.2)	0.255
Smoking history		192(58.0)	35(53.8)	0.535
Age(years old)		58.84 ± 10.27	60.23 ± 9.83	0.340
BMI(kg/m^2^)		25.37 ± 3.93	24.83 ± 4.39	0.349
Ejection fraction(%)		58.87 ± 10.83	56.12 ± 10.90	0.103
ApoB/ApoA1	High risk	9(2.7)	6(9.2)	0.023
	Low risk	322(97.3)	59(90.8)	
ApoB/ApoA1		0.91(0.71–1.04)	0.94(0.76–1.05)	0.152
TG(mmol/l)		1.73(1.0–2.13)	1.78(1.14–2.30)	0.267
LDL-c(mmol/l)		3.13(2.46–3.65)	3.17(2.46–3.70)	0.727
Non HDL-c(mmol/l)		3.5(2.77–4.10)	3.62(2.94–4.05)	0.464
Hb(g/L)		140.81(131–153)	140.5(129–155)	0.976
PLT(× 10^9^/L)		205.2(166–238)	193.01(157.5–227.5)	0.182
HCT(L/L)		0.93(0.39–0.45)	0.42(0.39–0.46)	0.768
LM		16(4.8)	16(24.6)	< 0.001
PLAD		216(65.3)	55(84.6)	< 0.001
MLAD		173(52.3)	38(58.5)	0.415
RCX		224(67.7)	56(86.2)	< 0.001
RCA		267(80.7)	59(90.8)	0.052
RAM		5(1.5)	2(3.1)	0.323
Number of lesions treated		1.38 ± 0.66	1.55 ± 0.73	0.032
Number of stents implanted		1.49 ± 0.90	1.59 ± 1.0	0.345
Maximum stent diameter(mm)		3.32 ± 0.49	3.24 ± 0.46	0.134

Compared with the low-risk group, the proportion of patients with LM, PLAD and RCX lesion were higher in the SYNTAX medium/high risk group (*p* < 0.001 respectively) (Table 1).

### Comparison of MACCE incidence and EQ-5D score at different time points between SYNTAX low and medium/high risk group

As shown in Table 2, compared with the low-risk group, SYNTAX medium/high risk group had higher MACCE rate in hospital (*p* = 0.049), and further significantly increased in 1 year and 3 years (*p* = 0.011, *p* = 0.023), while there was no statistical difference in MACCE rate in 5 years. The incidence of new or aggravated heart failure significantly increased in SYNTAX medium/high risk group after 1 year (*p* = 0.021), but there was no statistical difference in 3 and 5 years. Moreover, the rates of cardiovascular death, new myocardial infarction, revascularization and new stroke were similar between the two groups.

**Table 2:** Comparison of MACCE incidence at different time points between SYNTAX low and medium/high risk group [n(%)]

Item	Low-risk group (0–22)	Medium/high risk group (≥23)	*p*
MACCE events in hospital	19(4.4)	9(9.5)	0.049
MACCE events in 1 year	55(12.9)	22(23.2)	0.011
MACCE events in 3 years	112(26.2)	36(37.9)	0.023
MACCE events in 5 years	150(35.1)	41(43.2)	0.142
Cardiovascular death in hospital	10(2.3)	5(5.3)	0.165
Cardiovascular death in 1 year	20(4.7)	9(9.5)	0.081
Cardiovascular death in 3 years	36(8.4)	11(11.6)	0.325
Cardiovascular death in 5 years	50(11.7)	17(17.9)	0.125
New myocardial infarction in 1 year	5(1.2)	1(1.1)	1.000
New myocardial infarction in 3 years	14(3.3)	3(3.2)	1.000
New myocardial infarction in 5 years	25(5.9)	7(7.4)	0.578
Recurrent revascularization in 1 year	15(3.5)	2(2.1)	0.750
Recurrent revascularization in 3 years	33(7.7)	8(8.4)	0.820
Recurrent revascularization in 5 years	49(11.5)	13(13.7)	0.547
New/aggravated heart failure in 1 year	5(1.2)	5(5.3)	0.021
New/aggravated heart failure in 3 years	19(4.4)	7(7.4)	0.293
New/aggravated heart failure in 5 years	24(5.6)	8(8.4)	0.303
New stroke in 1 year	4(0.9)	1(1.1)	1.000
New stroke in 3 years	11(2.6)	3(3.2)	0.727
New stroke in 5 years	19(4.4)	4(4.2)	1.000

No significant difference of EQ-5D scores at different time points was seen between low-risk and medium/high risk group (Table 3).

**Table 3:** Comparison of EQ-5D score at different time points between SYNTAX low and medium/high risk group [M(P_25_-P_75_)]

Item	Low-risk group (0–22)	Medium/high risk group (≥23)	*P*
EQ-5D score in hospital	10.77(9.25–13.68)	11.03(9.40–13.82)	0.974
EQ-5D score in 1 year	12.99(12.23–14.52)	13.23(12.24–14.50)	0.677
EQ-5D score in 5 years	12.76(12.16–14.44)	12.88(12.16–14.44)	0.993

### Comparison of clinical baseline data between SYNTAX II low, medium and high risk group

Similarly, patients are divided into 3 groups according to SYNTAX II score [11, 12]: low-risk group (SYNTAX II score 0–21), medium-risk group (SYNTAX II score 22–28), high-risk group (SYNTAX II score ≥ 29).

Table 4 showed that except for the relevant clinical variables participating in the SYNTAX II scoring pattern, the proportion of patients with hypertension significantly increased in the medium-risk and high-risk group compared with the low-risk group (*p* = 0.003). In addition, apoB/apoA1 was more likely to be defined as high-risk in SYNTAX II medium-risk and high-risk group (*p* = 0.044). There was no statistical difference in the remaining general data and other serum biochemical items. Triglycerides significantly decreased in the other two groups compared with SYNTAX II low-risk group (*p* = 0.027), which may be related to the higher proportion of myocardial infarction and/or PCI history in this group thus the long-term adherence to the low-salt and low-fat diet prescribed by their physicians.

**Table 4:** Comparison of clinical baseline data among SYNTAX II low, medium and high-risk group [n(%),M(P_25_-P_75_)]

Item		Low-risk group (0–21)	Medium-risk group (22–28)	High-risk group (≥29)	*p*
n		103	156	137	
Gender	Male	99(96.1)	112(71.8)	63(46)	< 0.001
	Female	4(3.9)	44(28.2)	74(54)	
In-hospital ventricular fibrillation		0	1(0.6)	0	1.000
In-hospital ventricular tachycardia		1(1.0)	1(0.6)	1(0.7)	1.000
In-hospital atrial fibrillation		1(1.0)	4(2.6)	4(2.9)	0.637
In-hospital heart failure		12(11.7)	22(14.1)	36(26.3)	0.004
Hypertension		53(51.5)	113(72.4)	85(62.0)	0.003
Diabetes		18(17.5)	28(17.9)	33(24.1)	0.324
CHD history		17(16.5)	24(15.4)	20(14.6)	0.838
Myocardial infarction history		6(5.8)	8(5.1)	14(10.2)	0.201
PCI history		5(4.9)	6(3.8)	13(9.5)	0.109
Smoking history		73(70.9)	85(54.5)	69(50.4)	0.004
Age(years old)		51.10 ± 8.25	61.83 ± 7.50	66.63 ± 7.82	< 0.001
BMI(kg/m^2^)		25.79 ± 4.25	24.99 ± 3.27	24.91 ± 4.49	0.187
Ejection fraction(%)		60.95 ± 7.26	61.08 ± 9.53	51.81 ± 10.23	< 0.001
ApoB/ApoA1	High risk	2(1.9)	15(9.6)	8(5.8)	0.044
	Low risk	101(98.1)	141(90.4)	129(94.1)	
ApoB/ApoA1		0.88(0.69–1.01)	0.95(0.72–1.07)	0.89(0.71–1.04)	0.816
TG(mmol/l)		1.88(1.07–2.31)	1.85(1.04–2.26)	1.51(0.95–1.81)	0.027
LDL-c(mmol/l)		3.06(2..44–3.43)	3.19(2.49–3.74)	3.15(2.46–3.72)	0.372
Non HDL-c(mmol/l)		3.51(2.77–4.04)	3.59(2.85–4.23)	3.45(2.66–4.03)	0.465
Hb(g/L)		142.91(134–155)	141.03(131–153)	138.84(128–152)	0.249
PLT(×10^9^/L)		205.64(172–232)	202.76(158.25–239)	201.92(164.5–233)	0.937
HCT(L/L)		0.42(0.39–0.45)	0.42(0.38–0.45)	1.65(0.38–0.45)	0.370
LM		7(6.8)	12(7.7)	19(13.9)	0.124
PLAD		65(63.1)	110(70.5)	102(74.5)	0.165
MLAD		50(48.5)	87(55.8)	78(56.9)	0.397
RCX		64(62.1)	115(73.7)	110(80.3)	< 0.001
RCA		78(75.7)	134(85.9)	118(86.1)	0.086
RAM		2(1.9)	3(1.9)	0(0.0)	0.266
Number of lesions treated		1.31 ± 0.58	1.46 ± 0.71	1.50 ± 0.71	0.024
Number of stents implanted		1.42 ± 0.81	1.56 ± 0.97	1.56 ± 1.00	0.244
Maximum stent diameter(mm)		3.34 ± 0.51	3.30 ± 0.47	3.28 ± 0.47	0.584

Compared with the low-risk group, the proportion of patients with RCX lesion were higher in the SYNTAX II medium and high-risk group (*p* < 0.001) (Table 4).

### Comparison of MACCE incidence and EQ-5D score at different time points among SYNTAX II low, medium and high risk group

As shown in Table 5, compared with low-risk group, SYNTAX II medium and high-risk groups had higher MACCE incidence in 5 years (*p* = 0.032), significantly increased cardiovascular mortality in 3 and 5 years (*p* = 0.001, *p*<0.001 respectively), increased proportion of new or aggravated heart failure in 3 and 5 years (*p* = 0.015, *p* = 0.011 respectively). The incidence of myocardial infarction, revascularization and stroke was similar among these three groups.

**Table 5:** Comparison of MACCE incidence at different time among SYNTAX II low, medium and high-risk group [n(%)]

Item	Low-risk group (0–21)	Medium-risk group (22–28)	High-risk group (≥29)	*p*
MACCE events in hospital	5(3.3)	7(3.8)	16(8.6)	0.053
MACCE events in 1 year	19(12.6)	23(12.5)	35(18.7)	0.162
MACCE events in 3 years	35(23.2)	52(28.2)	61(32.8)	0.150
MACCE events in 5 years	41(27.2)	77(41.8)	73(39.2)	0.032
Cardiovascular death in hospital	2(1.3)	6(3.3)	7(3.8)	0.414
Cardiovascular death in 1 year	4(2.6)	12(6.5)	13(7.0)	0.175
Cardiovascular death in 3 years	6(4.0)	13(7.1)	28(15.1)	0.001
Cardiovascular death in 5 years	8(5.3)	18(9.8)	41(22.0)	< 0.001
New myocardial infarction in 1 year	1(0.7)	1(0.5)	4(2.1)	0.389
New myocardial infarction in 3 years	5(3.3)	3(1.6)	6(3.2)	0.211
New myocardial infarction in 5 years	9(6.0)	9(4.9)	14(7.5)	0.580
Recurrent revascularization in 1 year	6(4.0)	5(2.7)	6(3.2)	0.784
Recurrent revascularization in 3 years	15(9.9)	13(7.1)	13(7.0)	0.530
Recurrent revascularization in 5 years	22(5.3)	23(5.4)	17(9.1)	0.286
New/aggravated heart failure in 1 year	2(1.3)	2(1.1)	6(3.2)	0.314
New/aggravated heart failure in 3 years	3(2.0)	7(3.8)	16(8.6)	0.015
New/aggravated heart failure in 5 years	4(2.6)	9(4.9)	19(10.2)	0.011
New stroke in 1 year	2(1.3)	1(0.5)	2(1.1)	0.860
New stroke in 3 years	4(2.6)	5(2.7)	5(2.7)	1.000
New stroke in 5 years	5(3.3)	10(5.4)	8(4.3)	0.638

The baseline EQ-5D scores of SYNTAX II score groups showed a gradually decreasing trend, among which the high-risk group was the lowest (Table 6). The EQ-5D score in 1 year increased when compared with the baseline, but no statistical difference was observed among three groups, indicating that the short-term quality of life of the patients after PCI improved regardless of SYNTAX II score. Although the EQ-5D score in 5 years was higher than the baseline, it was still lower than the score in 1 year. The score of the high-risk group decreased significantly compared with the low and medium-risk group (with low-risk group, *p* = 0.019; with medium-risk group, *p* = 0.023), which meant the patients of the SYNTAX II high-risk group had a poor long-term quality of life.

**Table 6:** Comparison of EQ-5D score at different time among SYNTAX II low, medium and high-risk group [M(P_25_-P_75_)]

Item	Low-risk group (0–21)	Medium-risk group (22–28)	High-risk group (≥29)	*p*
EQ-5D score in hospital	11.58(10.0–13.81)	10.91(9.24–13.06)	9.90(9.2–13.76)	0.196
EQ-5D score in 1 year	13.28(12.24–14.93)	12.93(12.24–14.5)	12.85(12.16–13.82)	0.508
EQ-5D score in 5 years	13.19(12.22–15.03)	12.91(12.16–14.44)	12.08(10.64–12.92)	0.001

### Risk factors analysis of MACCE event incidence at different time points

After adjusting for potential confounding factors, multivariate logistic regression analysis showed in Table 7, 8 and 9. First, apoB/apoA1 (OR = 3.429, 95%CI: 1.264–12.672, *p* = 0.038) were independent predictors of in-hospital MACCE events (Table 7). Second, SYNTAX score was an independent predictor of MACCE events in 1 year, and the risk of MACCE events in SYNTAX medium-risk group was 2.124 times as that in the low-risk group (OR = 2.124, 95%CI: 1.124–4.013, *p* = 0.02), while the risk of MACCE events in SYNTAX high-risk group was 9.558 times as that in the low-risk group (OR = 9.558, 95%CI: 1.552–58.865, *p* = 0.015) (Table 8). Third, previous history of coronary heart disease (OR = 2.558, 95%CI: 1.053–6.215, *p* = 0.038), smoking (OR = 1.868, 95%CI: 1.026–3.402, *p* = 0.041), apoB/apoA1 (OR = 2.525, 95%CI:1.332–5.385, *p* = 0.016) and SYNTAX II score were independent predicers of MACCE events in 5 years, and the risk of MACCE events in SYNTAX II medium-risk group was 2.845 times as that in the low-risk group (OR = 2.845, 95%CI: 1.414–5.725, *p* = 0.003) (Table 9).

**Table 7:** Multivariate logistic regression analysis for in-hospital MACCE events

Factors	β value	S.E.	Wald χ^2^	*p*	OR value	OR 95%CI
ApoB/apoA1	0.939	0.453	4.303	0.038	2.558(1.053–6.215)	0.939

**Table 8:** Multivariate logistic regression analysis for MACCE events in 1 year

Factors		Control	*β* value	S.E.	Wald χ^2^	*p*	OR value	OR 95%CI
SYNTAX score	Medium-risk	Low-risk	0.753	0.325	5.387	0.02	2.124	1.124–4.013
	High-risk		2.257	0.927	5.924	0.015	9.558	1.552–58.865

**Table 9:** Multivariate logistic regression analysis for MACCE events in 5 years

Factors		Control	*β* value	*S.E.*	*Wald* χ^2^	*P*	*OR* value	OR 95%CI
Previous CHD history			0.939	0.453	4.303	0.038	2.558	1.053–6.215
Smoking			0.625	0.306	4.181	0.041	1.868	1.026–3.402
SYNTAX II score	Medium-risk	Low-risk	1.046	0.357	8.592	0.003	2.845	1.414–5.725
ApoB/apoA1			1.511	0.625	5.853	0.016	2.525	1.332–5.385

## Discussion

Clinical studies have found that the severity of coronary lesion is usually positively correlated with the severity of ACS. Therefore, it is recommended to use coronary angiography to calculate the coronary lesion score and then evaluate the severity of coronary lesion. A new scoring system called *SYNTAX* based on the anatomic characteristics of coronary arteries emerged in this context and played an important role in distinguishing the advantages and disadvantages of PCI or CABG in the treatment of complex lesions such as three-vessel lesions and/or left main lesions initially [13, 14]. Since then, more and more studies have focused on the predictive value of this scoring system for the prognosis of complex lesions. Brkovic et al. found that SYNTAX score was superior to GRACE risk score, TIMI blood flow grading score, PAMI score and ZWOLLE score in predicting MACE events and cardiovascular mortality [15]. He’s and other studies showed that in the use of the second generation of drug-eluting stents (DES) for the treatment of left main lesion patients, SYNTAX II score is an independent predictor of long-term mortality and has better predictive value than SYNTAX score [16]. For our study, we focused on the prognostic value of different SYNTAX scores in universal ACS patients.

The data showed that SYNTAX score was an independent predictor of the incidence of MACCE events in 1 year. The risk of MACCE events in SYNTAX medium-risk group was more than 1 times higher than the low-risk group while high-risk group was more than 8 times higher. However, no significant difference was observed in the risk of MACCE events in 5 years. Whereas SYNTAX II score had no statistical relationship with 1-year MACCE incidence, it was an independent predictor of the incidence of MACCE events in 5 years. The risk of MACCE events in SYNTAX II medium-risk group was more than 2 times higher than the low-risk group. It can be seen from the above results that the incidence of MACCE events in 1 year after coronary stenting is mostly correlated with angiographic features, while the incidence of MACCE events in 5 years after coronary stenting is more correlated with clinical features such as renal function and cardiac ejection function except for coronary artery lesions. That is, SYNTAX score has good predictive value of short-term prognosis, while SYNTAX II score is more predictive of long-term prognosis. The cardiovascular mortality in 3 and 5 years in SYNTAX II middle and high-risk group significantly increased whereas SYNTAX groups showed no significant difference, which means SYNTAX II score is superior to SYNTAX score in predicting cardiovascular death and is more suitable for medium and long-term prediction. The EQ-5D scores of different groups all showed the lowest baseline, the highest in 1 year, and the trend of decline in 5 years. Since the clinical follow-up observation is often limited to about 1 year when the quality of life of the patients improve compared with that of hospitalization, both the medical staff and patients are easy to relax their vigilance. In addition, the EQ-5D score in 5 years of SYNTAX II high-risk group significantly decreased compared with low and medium-risk group. This indicates that the long-term prognosis of SYNTAX II high-risk group is poor, so the clinical follow-up observation period should be extended, and the patients should be reminded to pay attention to relevant examination, removal and/or control of risk factors.

Since we included all patients who underwent stent implantation and did not differentiate between the types of ACS or lesions, the above conclusions are applicable to the universal ACS patients. This also led us to further consider that there were no statistically significant differences in common risk factors of coronary heart disease (including medical history, personal history and laboratory examination) in each group, why some patients have more serious coronary artery lesion while others not? Statistical analysis revealed a specific ratio, apoB/apoA1.

ApoB is a major apolipoprotein in the atherogenic lipoprotein family (VLDL, IDL, LDL, Lp (a), in which LDL is transformed from VLDL and IDL), which can reflect the total number of atherogenic lipoprotein particles [17]. LDL transports cholesterol to peripheral tissues and modifies it subcutaneously within the blood vessels to form oxidized LDL (ox-LDL), which is then ingested by macrophages to form foam cells [18]. Foam increase and fuse to form the lipid core of atherosclerotic plaques [19]. ApoA1 is the main apolipoprotein of HDL, which can reflect the total number of anti-atherosclerotic lipoproteins. HDL transports cholesterol from peripheral tissues to the liver for catabolism, reduces the deposition of cholesterol in the peripheral blood vessel wall, and plays an anti-atherosclerosis role. ApoB/apoA1 ratio is an indicator reflecting the balance of transport between atherosclerotic lipoprotein and anti-atherosclerotic lipoprotein in vivo. The increase of ApoB or decrease of apoA1 indicates the increase of cholesterol transport to peripheral tissues or decrease of cholesterol transport back to liver, leading to more cholesterol deposition on the blood vessel wall and promoting the occurrence of atherosclerosis (AS). Jung found that apoB/apoA1 level was positively correlated with the non-calcified plaque incidence, vascular stenosis rate [20]. Moreover, studies on Chinese Han population found that apoB/apoA1 was correlated with coronary heart disease risk factors such as diabetes mellitus and abnormal glucose tolerance [21]. ApoB/apoA1 can be used as a predictor of coronary heart disease risk [22, 23], but its effect on prognosis of ACS patients is rarely reported. Our data showed that, compared with the low-risk group, apoB/apoA1 was more likely to be defined as high risk in both SYNTAX and SYNTAX II medium and high-risk group. There was no statistical difference in mean apoB/apoA1 values, but the middle and high-risk groups were all higher than the low-risk groups. Multivariate logistic regression analysis showed that apoB/apoA1 was the predictor of MACCE events in hospital and in 5 years after discharge. It follows that apoB/apoA1 is positively correlated with the severity of coronary artery diseases and the prediction of long-term prognosis.

In conclusion, for universal ACS patients undergoing stent implantation, SYNTAX score has a high predictive value for short-term prognosis while SYNTAX II score is more predictive of long-term prognosis. SYNTAX II score is superior to SYNTAX score in predicting cardiovascular death. The combination of apoB/apoA1 high-risk and SYNTAX II medium and high-risk group is the focus of clinical treatment and long-term follow-up observation. At present, there is no uniform risk stratification standard for apoB/apoA1 internationally. In our study, the number of patients who were defined as apoB/apoA1 high risk was relatively small. For the next step, we intend to find the risk stratification standard and intervention target value suitable for Chinese people by expanding the sample size or setting coronary artery negative control group, so as to further reduce the mortality of high-risk ACS patients. The limitation of this study is that the stent selection bias was not excluded.

## Conclusions

Our study highlight the different prognosis value of SYNTAX and SYNTAX II score, which provides clinicians with a powerful tool for predicting short and long-term outcomes in universal coronary heart disease. We also emphasize which patients should be the focus of clinical treatment and long-term follow-up observation.

## Data Availability

All data generated or analysed during this study are included in this published article.

## Abbreviations

PCI: Percutaneous coronary intervention
MACCE: Major adverse cardiovascular and cerebrovascular events
CHD: Coronary heart disease
ACS: Acute coronary syndrome
CABG: Coronary artery bypass grafting
HCR: Hybrid coronary revascularization
TG: Triglyceride
TC: Total cholesterol
LDL-c: Low density lipoprotein-cholesterol
non-HDL-c: Non-high density lipoprotein-cholesterol
Hb: Hemoglobin
PLT: Platelet
HCT: Hematokrit
LM: Left main artery
PLAD: Left anterior descending ramus proximal segment
MLAD: Left anterior descending ramus middle and distal segment
RCX: Obtuse marginal,ramus circumflexus, left posterior descending ramus, left posterolateral ramus
RCA: Right coronary artery, right posterior descending ramus, right posterolateral ramus, acute marginal ramus
RAM: Ramus medianus
LVEF: Left ventricular ejection fraction
PVD: Peripheral vascular disease
COPD: Chronic obstructive pulmonary disease
CI: Confidence interval
